# LIFE EXTENSION VIA GENETIC/DRUG INTERVENTIONS OCCURS BY DELAYING THE ONSET OF AGING, NOT BY SLOWING THE RATE OF AGING

**DOI:** 10.1093/geroni/igae098.2294

**Published:** 2024-12-31

**Authors:** Patrick Phillips, Christine Sedore, Grace Jackson, Gordon Lithgow, Monica Driscoll

**Affiliations:** University of Oregon, Eugene, Oregon, United States; University of Oregon, Eugene, Oregon, United States; University of Oregon, Eugene, Oregon, United States; Buck Institute for Research on Aging, Navado, California, United States; Rutgers, The State University of New Jersey, Piscataway, New Jersey, United States

## Abstract

Ever since the discovery more than 30 years ago of mutations capable dramatically increasing lifespan, there has been an increasing push to find genetic pathways and chemical interventions that ameliorate the effects of aging and increase healthspan and longevity. But aging is not merely “getting old,” it is an accelerating rate of the decline in function and an increase in the rate of mortality with chronological age. Here, we present a new framework for understanding longevity-extending interventions by dividing the survivorship into two major parts—the onset of aging and the period of accelerated aging—and use a uniform analytical approach to derive these quantities for each of the major mortality models. We apply this new framework to a set of studies with sufficient size to estimate mortality rate, finding that for four genetic and three chemical interventions in C. elegans and five genetic and eleven chemical interventions in mice, all treatments that lead to an increase in overall lifespan do so by delaying the onset of aging and none decrease the rate of aging late in life. These effects are readily visualized using a relative hazard and relative lifespan approach. Thus, while there is great interest identify compounds and other approaches to “treat” aging, evidence to date suggests that these interventions do not actually affect aging in the formal sense. These results have important implications for the design and analysis of aging studies, as well as for the field of geroscience as a whole.

